# Supplementary material for: Integrating stereotypes and factual evidence in interpersonal communication
Source: NPJ Sci Learn. 2024 Aug 22;9:52. doi: 10.1038/s41539-024-00262-6 (PMC11341559; doi:10.1038/s41539-024-00262-6)
Supplement: Supplementary file 1 — Supplementary Information [file 41539_2024_262_MOESM1_ESM.pdf]

Supplementary Information

Integrating stereotypes and factual evidence in interpersonal communication

Saskia B. J. Koch, Anna Tyborowska, Hannah C. M. Niermann, Antonius H. N. Cillessen, Karin Roelofs, Jana Bašnáková, Ivan Toni & Arjen Stolk

Supplementary Results

In the following sections, we report a number of additional analyses designed to verify the specificity and robustness of the findings reported in the main text.

Communicative specificity of the stereotype-based adjustments. We performed additional analyses to verify whether the stereotype-driven adjustments reported in the main analysis (Figure 1B) were specific to communicatively relevant portions of the game board and did not influence other aspects of participants' behavior such as planning time and time spent in other locations of the game board. We report the results of Bayesian linear mixed models, considering planning time and time spent in other locations as dependent variables, presumed partner (adult, child) as a within-subject factor, and participant as a random factor to account for individual differences. We used the *brms* package in R with default priors for all models[1]. We considered effects to be statistically significant in case the 95% credible interval (CI) of the posterior distributions did not contain zero. Additionally, posterior probability (*pp*) values are reported, which can be interpreted as traditional *p*-values at an alpha level of 0.025[2]. The models found that the dependent variables of participants' planning time (B = -0.01, 95% CI = [-0.03 0.01], *pp* = 0.143) and time spent in other locations of the game board (Supplementary Figure 1; B = -0.01, 95% CI = [-0.03 0.01], *pp* = 0.191) were matched between both presumed partners. Moreover, a cluster-based analysis found no communicative adjustments in other locations of the game board across any partner transition. These results support the notion that the stereotype-driven adjustments were specific to communicatively relevant aspects of participant behavior.

Consistency of the confederate's task performance. We performed additional analyses to determine if the confederate showed consistent behavior and understanding in the roles of the child and adult partner. We report the results of Bayesian (generalized) linear mixed models using the same specifications as above, but with the proportion of correctly solved trials and the response time of the confederate as dependent variables. The results of the analysis showed that the proportion of correctly solved trials (Supplementary Figure 2a; B = 0.09, 95% CI = [-0.03 0.22], *pp* = 0.075) and the confederate's response time (Supplementary Figure 2c; B = 0.01, 95% CI = [-0.01 0.04], *pp* = 0.124) were matched between both partners. An additional analysis focused on the consistency of the confederate's performance across participants found that there was no statistically significant relationship between the order in which the participants were tested and the proportion of correctly solved trials (Supplementary Figure 2b; $\rho_{(95)} = 0.08$, $p = 0.420$) or confederate's response time (Supplementary Figure 2d; $\rho_{(95)} = 0.10$, $p = 0.340$). These results suggest that the confederate's task performance was consistent within and across participants.

Generalizability of the structural brain-behavior findings. We performed additional analyses to assess the generalizability of the identified association between the right anterior cingulate gyrus (ACCg) and stereotype-driven communicative adjustment (Figure 2a). We used two additional datasets, including the same sample at age 14 (45 individuals; 23 females) and an independent sample that performed the same task (27 individuals; 27 males; 22.67 ± 3.41 y of age). For the participants at age 14, identical systems and protocols were used to acquire the T1-weighted MRI data. For the independent sample, structural images were acquired with a 1.5T Siemens Avanto scanner using a single-shot MPRAGE sequence (TR/TE, 1.73 s/2.95 ms; voxel size, 1 x 1 x 1 mm^3; FOV, 256 mm). This dataset comprised the placebo group from a study on the effects of intranasal oxytocin administration on communicative adjustment[3,4]. We applied the same voxel-based morphometry pipeline as used in the main analysis to both datasets, and extracted right ACCg volume using a statistical mask based on the findings reported in the main text. For the participants at age 14, we correlated right ACCg volume with communicative adjustment at age 17. For the independent sample, we correlated right ACCg volume with communicative adjustment during partner transition 6 through 8, when adjustment was statistically prominent[3]. As shown in Supplementary Figure 4, Spearman rank correlations found that communicative adjustment was positively associated with volume in the right ACCg in both the same sample at age 14 ($\rho_{(45)} = 0.376$, $p = 0.011$) and the independent sample ($\rho_{(27)} = 0.389$, $p = 0.045$). Moreover, even with a strong correlation between right ACCg volume at age 14 and the corresponding volume at age 17 ($\rho_{(42)} = 0.887$, $p < 0.001$), the association with stereotype-driven adjustment at age 17 remained significant after adjusting for right ACCg volume at age 14 ($\rho_{(36)} = 0.389$, $p = 0.016$). These

results suggest the timeliness and replicability of the structural brain-behavior relationships highlighted in the main analysis across various datasets.

Specificity and persistence of the effects of early social experience. We conducted additional analyses to ascertain whether the effects of early social experiences on communicative adjustment (Figure 2c) retained significance after accounting for the effects of familial environment (parents' socio-economic status and the presence of siblings), late social experiences (number of friends at age 7, extracurricular activities at age 12, and time spent with friends at age 14), and participants' education levels (at age 16). We used partial Spearman rank correlation analyzes to control for variance accounted for by these social environmental factors and found that the inverse relationship between daycare attendance and communicative adjustment remained statistically significant. Moreover, daycare attendance did not covary with any of the late social experiences (all $\rho \leq 0.169$), and as seen in Supplementary Figure 6, was the strongest predictor of communicative adjustment at age 17. These results suggest that the effects of early social experiences on communicative adjustment are specific and persistent.

Communicative specificity of the interaction-based adjustments. We performed additional analyses to verify whether the effects of early social experiences on communicative adjustment were specific to communicatively relevant portions of the game board and did not influence other aspects of participant behavior, including planning time and time spent in other locations of the game board. We report the results of Bayesian linear mixed models, considering planning time and time spent in other locations as dependent variables, presumed partner (adult, child) as a within-subject factor, participant as a random factor, and daycare attendance and familial environment as predictors. We also modeled the interaction between these predictors and the factor of partner. We found that daycare attendance was positively associated with planning time (B = 0.10, 95% CI = [0.01 0.19], *pp* = 0.017), but that this relationship was not modulated by partner (B = -0.01, 95% CI = -0.03 0.01, *pp* = 0.313). Daycare attendance was not associated with other indices of task performance such as proportion of correctly solved trials (B = -0.06, 95% CI = [-0.35 0.23], *pp* = 0.337) and time spent in other locations of the game board (B = 0.11, 95% CI = [-0.01 0.24], *pp* = 0.037). Additionally, time spent in daycare did not modulate the relationship between partner and the proportion of correctly solved trials (B = -0.00, 95% CI = [-0.12 0.11], *pp* = 0.480) or time spent in other visited locations (B = 0.00 95% CI = [-0.01 0.02], *pp* = 0.285). Moreover, a cluster-based analysis found no significant association between daycare attendance and communicative adjustments in other locations across any partner transition, nor was the rate of convergence in time spent in these other locations significantly

associated with daycare attendance ($\rho_{(91)}$ = 0.018, p = 0.862). Finally, we investigated whether the effects of early social experiences on communicative adjustment could be explained by varying sensitivities to feedback among participants. A Bayesian generalized linear mixed model considering success as the dependent variable and daycare attendance, trial number, and presumed partner as predictors found no statistically significant relationship between presumed partners and the dynamics of success (B = -0.00, 95% CI = [-0.02 0.01], *pp* = 0.341). Moreover, there was no statistically significant interaction between daycare attendance, trial number, and partner (B = -0.00, 95% CI = [-0.01 0.01], *pp* = 0.311). Together, these results suggest that the effects of daycare attendance on communicative adjustment were specific to communicatively relevant portions of the game board and not influenced by other factors.

Supplementary Figures

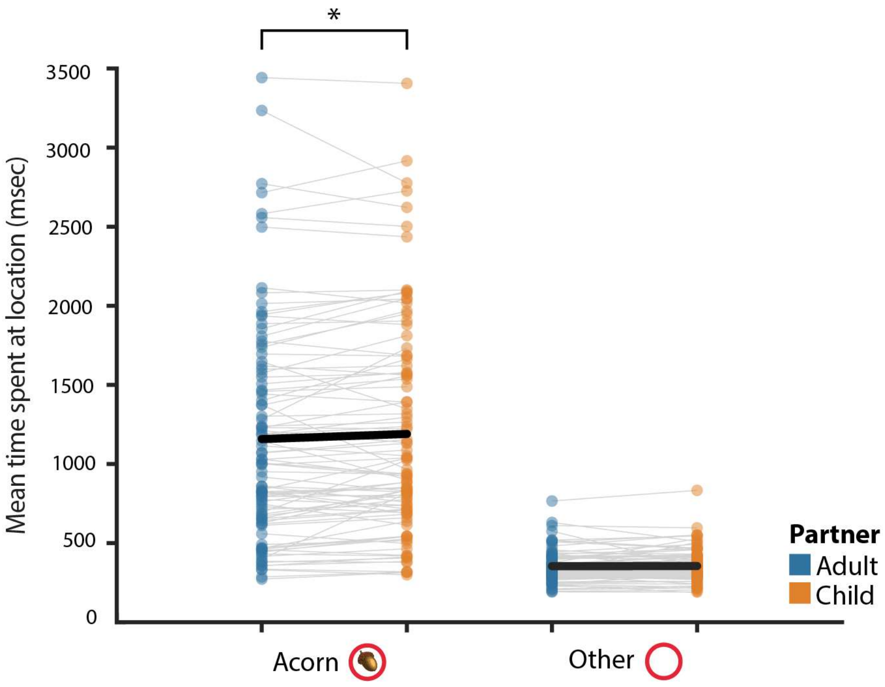

Supplementary Figure 1. Average time spent in different locations of the game board as a function of the presumed partner. Participants spent more time in the location of the acorn when interacting with the child partner compared to the adult partner (*M difference* = 31.58 ms, $t_{(94)}$ = 2.31, p = 0.023, Cohen's d = 0.24). There was no significant difference in the time spent in other locations between the two presumed partners (*M difference* = -3.46 ms, $t_{(94)}$ = -0.69, p = 0.491, Cohen's d = 0.07). Dots in the graph represent individual data points and the black lines indicate averages.

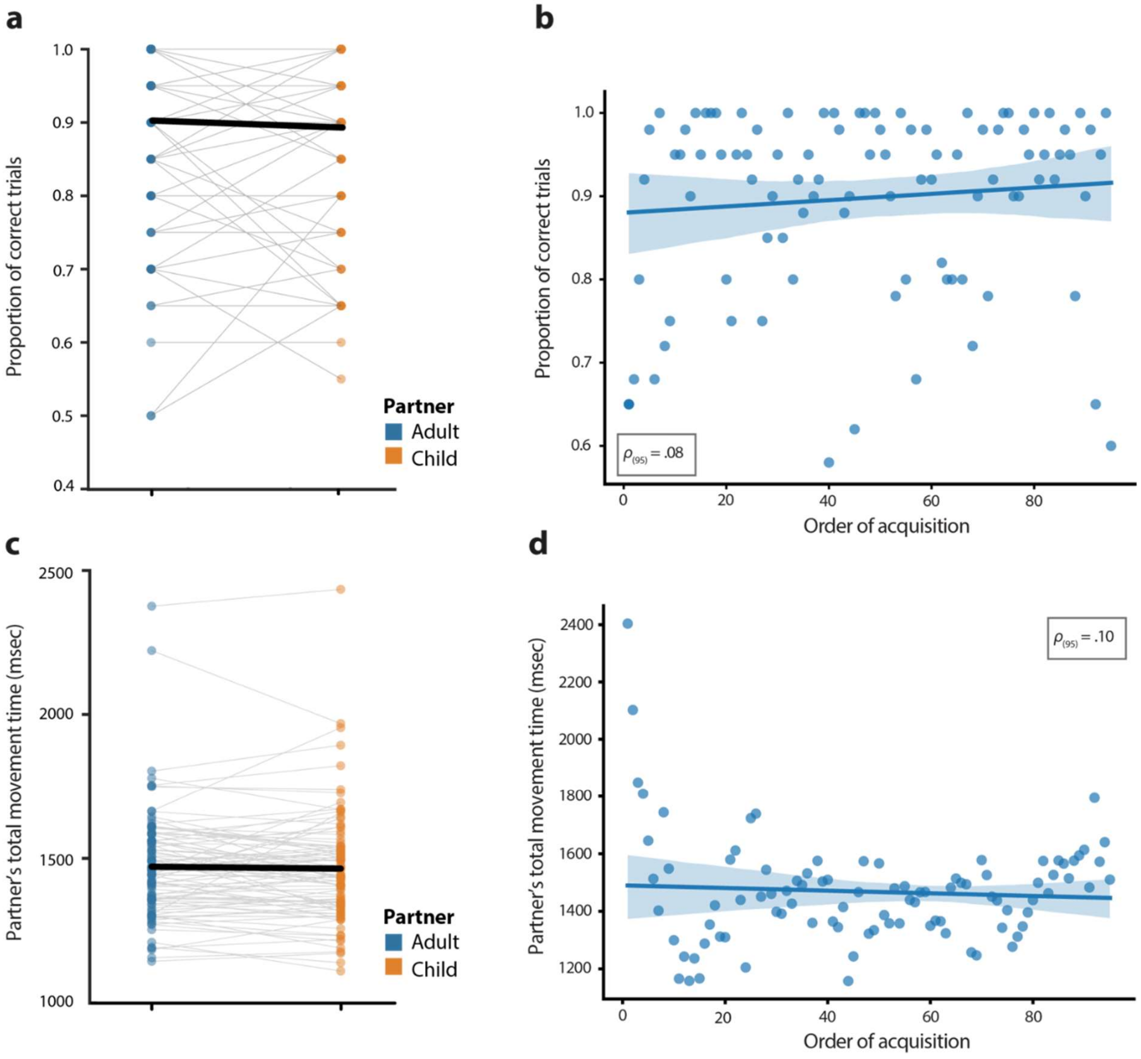

Supplementary Figure 2. Consistency of the confederate's task performance both within and across participants. (**a**) The proportion of correctly solved trials was consistent between the adult and child partner within each participant, and (**b**) this consistency was maintained across participants and did not change as a function of the order in which participants were tested. (**c**) The confederate's response time was consistent between both partners within each participant, and (**d**) this consistency did not change as a function of the acquisition order.

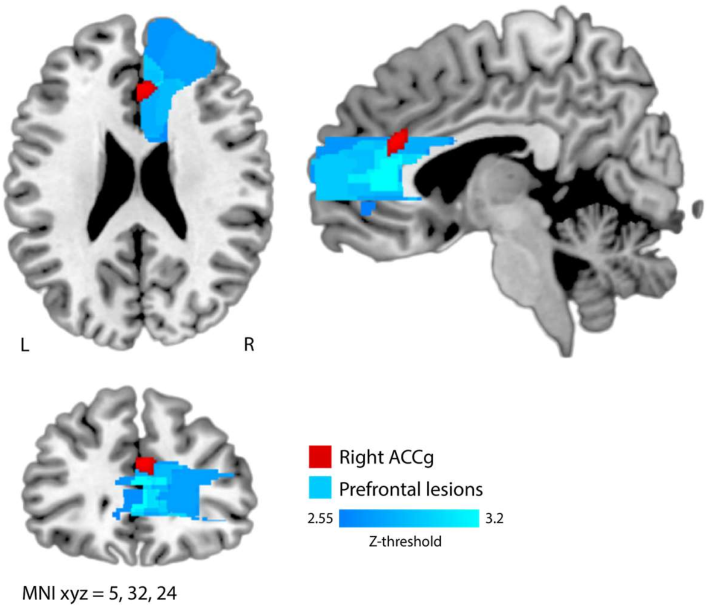

Supplementary Figure 3. Anatomical overlay of the cluster in the anterior cingulate gyrus (ACCg) found to be associated with stereotype-driven communicative adjustment (red, from Figure 2a) on a lesion-based voxelwise map of prefrontal brain regions that are crucial for adjusting communication based on stereotype beliefs (blue, adapted from[5]). Z-values correspond to a significance level of $0.05 > p > 0.001$ (two-tailed).

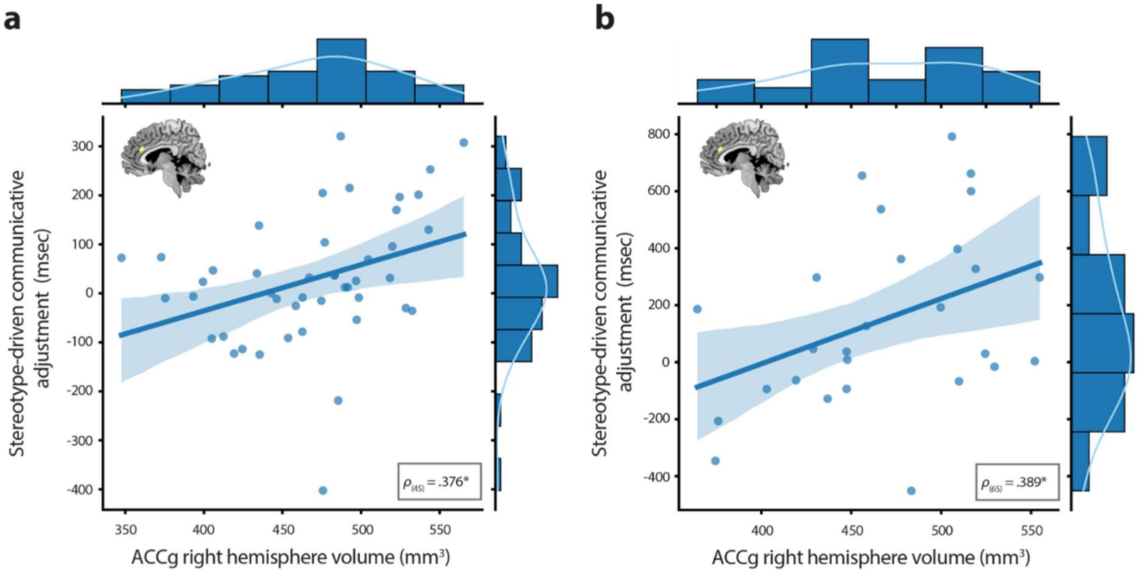

Supplementary Figure 4. Replication of the association between gray matter density in the anterior cingulate gyrus (ACCg) and communicative adjustment to stereotype beliefs in two additional datasets, namely (**a**) the same participants at age 14 and (**b**) an independent sample performing the same task.

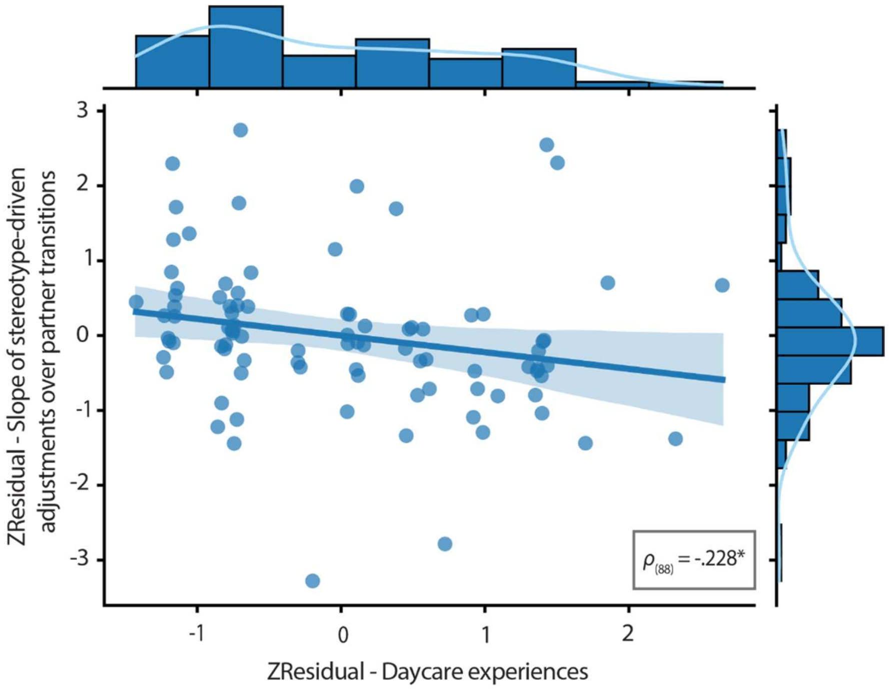

Supplementary Figure 5. Impact of early social experiences on the rate of communicative convergence at age 17. Time spent in daycare was predictive of the slope's steepness derived from the temporal dynamics of (declining) stereotype-driven communicative adjustment. ZResidual = standardized residual, corrected for the intercept of the linear function fitted through partner transitions.

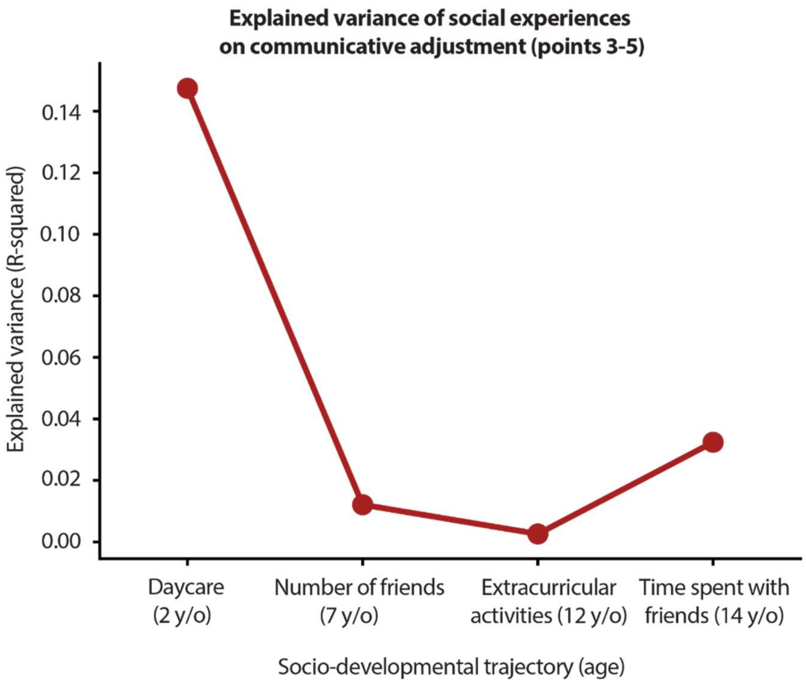

Supplementary Figure 6. Effects of early and late social experiences on interaction-based communicative adjustment at age 17. Time spent in daycare around the second year of life accounted for over 14% of the variance in (reduced) stereotype-driven communicative adjustment. By contrast, late social experiences (number of friends at age 7, extracurricular activities at age 12, and time spent with friends at age 14) did not significantly contribute to the variance in interaction-based communicative adjustment.

References

1. Bürkner, P.-C. brms: An R Package for Bayesian Multilevel Models Using Stan. *J. Stat. Softw.* **80**, 1–28 (2017).
2. Makowski, D., Ben-Shachar, M. S., Chen, S. H. A. & Lüdecke, D. Indices of Effect Existence and Significance in the Bayesian Framework. *Front. Psychol.* **10**, 2767 (2019).
3. Liu, R., Stolk, A., Boer, M. de, Oostenveld, R. & Toni, I. Oxytocin facilitates communicative adjustment by upregulating broadband aperiodic neural activity. Preprint at https://doi.org/10.31234/osf.io/kq9g6 (2021).
4. de Boer, M. *et al.* Oxytocin modulates human communication by enhancing cognitive exploration. *Psychoneuroendocrinology* **86**, 64–72 (2017).
5. Stolk, A., D'Imperio, D., di Pellegrino, G. & Toni, I. Altered Communicative Decisions following Ventromedial Prefrontal Lesions. *Curr. Biol.* **25**, 1469–1474 (2015).
