# Supplementary material for: Integrating stereotypes and factual evidence in interpersonal communication
Source: NPJ Sci Learn. 2024 Aug 22;9:52. doi: 10.1038/s41539-024-00262-6 (PMC11341559; doi:10.1038/s41539-024-00262-6)
Supplement: Supplementary file 2 — Reporting Summary [file 41539_2024_262_MOESM2_ESM.pdf]

nature portfolio

Corresponding author(s): Saskia B.J. Koch

Last updated by author(s): Dec 8, 2023

Reporting Summary

Nature Portfolio wishes to improve the reproducibility of the work that we publish. This form provides structure for consistency and transparency in reporting. For further information on Nature Portfolio policies, see our Editorial Policies and the Editorial Policy Checklist.

Statistics

For all statistical analyses, confirm that the following items are present in the figure legend, table legend, main text, or Methods section.

n/a	Confirmed	
☐	☒	The exact sample size (*n*) for each experimental group/condition, given as a discrete number and unit of measurement
☐	☒	A statement on whether measurements were taken from distinct samples or whether the same sample was measured repeatedly
☐	☒	The statistical test(s) used AND whether they are one- or two-sided<br>*Only common tests should be described solely by name; describe more complex techniques in the Methods section.*
☐	☒	A description of all covariates tested
☐	☒	A description of any assumptions or corrections, such as tests of normality and adjustment for multiple comparisons
☐	☒	A full description of the statistical parameters including central tendency (e.g. means) or other basic estimates (e.g. regression coefficient) AND variation (e.g. standard deviation) or associated estimates of uncertainty (e.g. confidence intervals)
☐	☒	For null hypothesis testing, the test statistic (e.g. *F*, *t*, *r*) with confidence intervals, effect sizes, degrees of freedom and *P* value noted<br>*Give P values as exact values whenever suitable.*
☐	☒	For Bayesian analysis, information on the choice of priors and Markov chain Monte Carlo settings
☒	☐	For hierarchical and complex designs, identification of the appropriate level for tests and full reporting of outcomes
☐	☒	Estimates of effect sizes (e.g. Cohen's *d*, Pearson's *r*), indicating how they were calculated

Our web collection on statistics for biologists contains articles on many of the points above.

Software and code

Policy information about availability of computer code

Data collection	The task was administered with Presentation Software (Neurobehavioral Systems, Albany, CA, USA).
Data analysis	Behavioral data were analyzed with Matlab (version 2022b) and R (version 4.2.3) in RStudio. Structural MRI data were analyzed with Matlab (version 2022b) and SPM12's computational anatomy toolbox (CAT12). Supplementary Bayesian analyses of behavioral data were conducted with the brms package, implemented in R (version 4.2.3).

For manuscripts utilizing custom algorithms or software that are central to the research but not yet described in published literature, software must be made available to editors and reviewers. We strongly encourage code deposition in a community repository (e.g. GitHub). See the Nature Portfolio guidelines for submitting code & software for further information.

Data

Policy information about availability of data

All manuscripts must include a data availability statement. This statement should provide the following information, where applicable:

- Accession codes, unique identifiers, or web links for publicly available datasets
- A description of any restrictions on data availability
- For clinical datasets or third party data, please ensure that the statement adheres to our policy

The datasets generated and analyzed during the current study will be made available in the Donders Repository (https://data.donders.ru.nl).

Research involving human participants, their data, or biological material

Policy information about studies with human participants or human data. See also policy information about sex, gender (identity/presentation), and sexual orientation and race, ethnicity and racism.

Reporting on sex and gender	Sex of the participants was determined based on parental report. In all structural MRI analyses, we control for participant's sex by including this factor as a covariate to the model.
Reporting on race, ethnicity, or other socially relevant groupings	Familial environment was indexed with parents' socio-economic status and the number of siblings. The former considered the average education level of both parents, sampled at 15 months of age. Because parental education level and daycare attendance were correlated, parental education level was residualised against the working hours of the mother to avoid multicollinearity in the statistical models.
Population characteristics	Participants were 17 year old adolescents. They were screened for any contra-indications to MRI (including a history of psychiatric or neurological disorders).
Recruitment	Participants were recruited through the Nijmegen Longitudinal Study on Infant and Child Development. Participants and their parents were first given written information about the study. Once participants showed interest to participate in the study, the general aims, methods, benefits, and potential risks of the study will be explained, and participants were screened for any contra-indications to MRI (including a history of psychiatric or neurological disorders). Written informed consent was obtained by the investigator from the participant and his/her parent/legal guardian.
Ethics oversight	The local ethics committee approved the study (CMO region Arnhem-Nijmegen, The Netherlands)

Note that full information on the approval of the study protocol must also be provided in the manuscript.

Field-specific reporting

Please select the one below that is the best fit for your research. If you are not sure, read the appropriate sections before making your selection.

☐ Life sciences ☒ Behavioural & social sciences ☐ Ecological, evolutionary & environmental sciences

For a reference copy of the document with all sections, see nature.com/documents/nr-reporting-summary-flat.pdf

Behavioural & social sciences study design

All studies must disclose on these points even when the disclosure is negative.

Study description	Longitudinal developmental study (quantitative).
Research sample	Healthy human volunteers (aged 16.5 - 17.5) who are participants of the Nijmegen Longitudinal Study.
Sampling strategy	Participants were recruited through the Nijmegen Longitudinal Study on Infant and Child Development. Given the uniqueness of the study and richness of the data collected, we aimed to include all participants from the NLS that were willing to participate. Based on analyses of fMRI sample size (e.g. Desmond & Glover, 2002), it was expected that 20-25 individuals per gender group is sufficient for a reliable within group average fMRI data at p<.05 cluster corrected. This suggest that our sample of 95 participants is sufficient.
Data collection	The communication game was administered with Presentation Software on a Windows XP computer. The structural MRI images were collected with 3Tesla Siemens MRI scanners. An experimenter stayed with the participant during the entire procedure.
Timing	July 2014 - April 2015
Data exclusions	One participant experienced difficulties adhering to the task instructions and was excluded from further analysis. Another participant was excluded because of poor MRI scan quality, leaving seventy individuals for structural brain-behavior analysis
Non-participation	128 infants started in the first wave of the Nijmegen Longitudinal Study (NLS). Of these, 96 participants completed the 10th wave when they were 17 years old. Participants mainly dropped out because they lost interest in study participation.
Randomization	n/a

Reporting for specific materials, systems and methods

We require information from authors about some types of materials, experimental systems and methods used in many studies. Here, indicate whether each material, system or method listed is relevant to your study. If you are not sure if a list item applies to your research, read the appropriate section before selecting a response.

Materials & experimental systems

n/a	Involved in the study
☒	☐ Antibodies
☒	☐ Eukaryotic cell lines
☒	☐ Palaeontology and archaeology
☒	☐ Animals and other organisms
☒	☐ Clinical data
☒	☐ Dual use research of concern
☒	☐ Plants

Methods

n/a	Involved in the study
☒	☐ ChIP-seq
☒	☐ Flow cytometry
☐	☒ MRI-based neuroimaging

Plants

Seed stocks

Report on the source of all seed stocks or other plant material used. If applicable, state the seed stock centre and catalogue number. If plant specimens were collected from the field, describe the collection location, date and sampling procedures.

Novel plant genotypes

Describe the methods by which all novel plant genotypes were produced. This includes those generated by transgenic approaches, gene editing, chemical/radiation-based mutagenesis and hybridization. For transgenic lines, describe the transformation method, the number of independent lines analyzed and the generation upon which experiments were performed. For gene-edited lines, describe the editor used, the endogenous sequence targeted for editing, the targeting guide RNA sequence (if applicable) and how the editor was applied.

Authentication

Describe any authentication procedures for each seed stock used or novel genotype generated. Describe any experiments used to assess the effect of a mutation and, where applicable, how potential secondary effects (e.g. second site T-DNA insertions, mosiacism, off-target gene editing) were examined.

Magnetic resonance imaging

Experimental design

Design type

Structural T1-weighted MRI data

Design specifications

n/a (a single T1-weighted scan was acquired)

Behavioral performance measures

n/a (The behavioral task (communication game) was acquired in a separate behavioral session, not during fMRI scanning).

Acquisition

Imaging type(s)

T1-weighted structural scan

Field strength

3 Tesla

Sequence & imaging parameters

MP-RAGE sequence (TR/TE, 2.30 s/3.03 ms; voxel size, 1 x 1 x 1 mm3; FOV, 256 mm)

Area of acquisition

Whole-brain scan

Diffusion MRI ☐ Used ☒ Not used

Preprocessing

Preprocessing software

SPM12's computational anatomy toolbox, CAT12

Normalization

After segmenting the anatomical images into gray matter, white matter, and cerebrospinal fluid, they were coregistered to MNI space using DARTEL.

Normalization template

A group specific template generated by DARTEL

Noise and artifact removal

n/a

Volume censoring

n/a

Statistical modeling & inference

Model type and settings

Multiple linear regression model

Effect(s) tested: The association between stereotype driven communicative adjustment and 1) gray matter asymmetry and 2) cortical thickness with a linear regression analysis.

Specify type of analysis: ☐ Whole brain ☐ ROI-based ☒ Both

Anatomical location(s): right anterior cingulate gyrus (ACCg)

Statistic type for inference (See Eklund et al. 2016): Threshold-free cluster enhancement (TFCE) method (p family wise error corrected < .05)

Correction: Family-wise error correction (FWE)

Models & analysis

n/a	Involved in the study
☒	☐ Functional and/or effective connectivity
☒	☐ Graph analysis
☒	☐ Multivariate modeling or predictive analysis
